# The peroxisomal multifunctional protein interacts with cortical microtubules in plant cells

**DOI:** 10.1186/1471-2121-6-40

**Published:** 2005-11-28

**Authors:** Simon DX Chuong, Nam-Il Park, Michelle C Freeman, Robert T Mullen, Douglas G Muench

**Affiliations:** 1 Department of Biological Sciences, University of Calgary, 2500 University Dr. NW, Calgary, AB, Canada T2N 1N4, Canada; 2 Department of Molecular and Cellular Biology, University of Guelph, Guelph, ON, Canada N1G 2W1, Canada

## Abstract

**Background:**

The plant peroxisomal multifunctional protein (MFP) possesses up to four enzymatic activities that are involved in catalyzing different reactions of fatty acid β-oxidation in the peroxisome matrix. In addition to these peroxisomal activities, *in vitro *assays revealed that rice MFP possesses microtubule- and RNA-binding activities suggesting that this protein also has important functions in the cytosol.

**Results:**

We demonstrate that MFP is an authentic microtubule-binding protein, as it localized to the cortical microtubule array *in vivo*, in addition to its expected targeting to the peroxisome matrix. MFP does not, however, interact with the three mitotic microtubule arrays. Microtubule co-sedimentation assays of truncated versions of MFP revealed that multiple microtubule-binding domains are present on the MFP polypeptide. This indicates that these regions function together to achieve high-affinity binding of the full-length protein. Real-time imaging of a transiently expressed green fluorescent protein-MFP chimera in living plant cells illustrated that a dynamic, spatial interaction exits between peroxisomes and cortical microtubules as peroxisomes move along actin filaments or oscillate at fixed locations.

**Conclusion:**

Plant MFP is associated with the cortical microtubule array, in addition to its expected localization in the peroxisome. This observation, coupled with apparent interactions that frequently occur between microtubules and peroxisomes in the cell cortex, supports the hypothesis that MFP is concentrated on microtubules in order to facilitate the regulated import of MFP into peroxisomes.

## Background

Peroxisomes are single-membrane-bound organelles that lack a genome and, therefore, must import their entire complement of constituent proteins. All proteins that are targeted to the peroxisome are synthesized on free polyribosomes in the cytosol and are imported post-translationally. Several distinct import pathways exist for membrane and matrix proteins (reviewed in [1]). For example, peroxisomal membrane proteins can be targeted either directly to the peroxisome from their sites of synthesis in the cytosol, or indirectly to peroxisomes via the endoplasmic reticulum ([2,3], and references therein). On the other hand, peroxisomal matrix proteins can be imported from the cytosol in their fully-folded conformation and as oligomeric protein complexes. Two types of peroxisomal matrix protein import pathways have been identified and well characterized [4,5]. Most matrix-destined proteins possess a type 1 peroxisomal targeting sequence (PTS1) that consists of an uncleaved carboxyl-terminal tripeptide sequence (small, basic and hydrophobic amino acids or a variant thereof). The cognate receptor for PTS1-bearing proteins, peroxin 5 (Pex5p), is proposed to carry its protein cargo into the peroxisome matrix as it cycles between the cytosol and the matrix. Alternatively, it may release its cargo after docking with the import machinery located on the peroxisomal surface [6]. In contrast to the PTS1, the type 2 PTS (PTS2) is located near the amino terminus of a smaller set of peroxisomal matrix-destined proteins. In plants and mammals the PTS2 is proteolytically cleaved following import. The receptor protein for PTS2 targeted proteins, Pex7p, is probably best characterized in yeast cells where it is proposed to cycle in and out of peroxisomes, similar to its Pex5p counterpart [7]. Pex7p also relies on Pex5p for the import of PTS2-containing proteins, indicating that the two matrix protein pathways are coupled [8,9].

In all organisms examined to date, peroxisomes are remarkably dynamic in terms of their shapes and intracellular movements [10-15]. Peroxisome morphology ranges from spherical and dumbbell-shaped, to extensively elongated and reticulated. Peroxisome movement and distribution is also highly variable and is mediated by either MTs or actin filaments, depending on the species. For instance, mammalian peroxisomes utilize a MT-based system for movement that is directed by dynein/dynactin and possibly kinesin motors [13]. In plant and yeast cells, actin filaments serve as the tracks for peroxisome movement, and myosin motors have been reported to be responsible for this movement [11,14,16-18]. Examples of actin-based plant peroxisome movements include rapid oscillations at fixed locations, stop-and-go movements in forward and reverse directions, and rapid longer distance movements that achieve velocities of up to 10 μm per second (for review, see [10]).

While MT involvement has not been reported for the directed motility of peroxisomes in plant cells, recent evidence indicates that MTs play a role in peroxisome protein import. Specifically, the peroxisomal multifunctional protein (MFP) has been shown to possess MT-binding activity *in vitro *[19]. MFP is a PTS1-containing peroxisomal matrix protein that possesses up to four enzymatic activities involved in catalyzing different reactions of the fatty acid β-oxidation pathway [20,21]. We proposed that the MT-binding activity of MFP serves to concentrate MFP in order to improve the efficiency of its import into peroxisomes [10]. This would necessitate that peroxisomes frequently be in close proximity to MTs in order for MT-bound MFP to interact with the import machinery located on the peroxisomal surface. Indirect evidence supporting a general role for MTs in peroxisomal protein import has come from a recent large scale proteomic study demonstrating the *in vitro *tubulin binding activity of five additional plant peroxisomal matrix proteins [22]. Although there is increasing biochemical evidence for MT-binding activities associated with plant peroxisomal matrix proteins, there have been no reports for this interaction *in vivo*.

Here we demonstrate that the peroxisomal MFP is an authentic MT-binding protein that associates specifically with interphase cortical MTs, but not with the MT arrays that form during cell division. We also present deletion analysis data indicating that multiple MT-binding domains are present on the MFP polypeptide. Additionally, live-cell imaging of a GFP-MFP chimera that localized to both peroxisomes and MTs revealed that peroxisomes are frequently in close association with MTs in the cell. This suggests that peroxisomes interact with cortical MTs as they move along actin filaments or oscillate at fixed locations. Overall, the localization of MFP to cortical MTs *in situ *and the apparent cortical MT-peroxisome interactions support the hypothesis that MTs have an important role in the targeting of MFP to peroxisomes.

## Results

### MFP localizes to both peroxisomes and cortical MTs in onion epidermal cells

**Figure 1:**
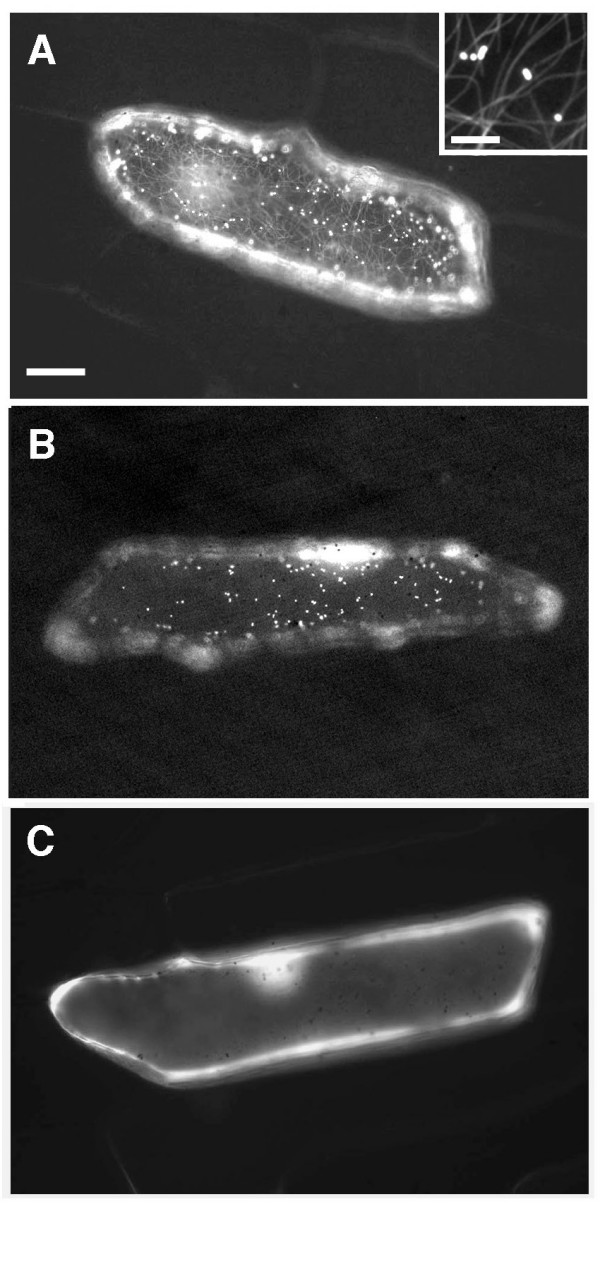
**Expression of a GFP-MFP fusion protein in onion epidermal cells labels peroxisome-like structures and MT-like filaments**. Onion epidermal layers were bombarded with DNA constructs encoding either GFP-MFP (A), GFP containing a carboxyl-terminal PTS1 (GFP-SRM) (B), or GFP alone (C), and visualized by epifluorescence microscopy. The inset in (A) shows a representative confocal image of peroxisome-like structures and MT-like filaments in the same optical Z-section in the cortical region of a GFP-MFP-expressing cell. Bars, 20 μm and 3 μm (A, inset).

To begin to examine the putative interaction between MFP and MTs in plant cells, an expression construct encoding GFP fused to the amino terminus of MFP (GFP-MFP) was introduced into onion epidermal cells by particle bombardment. Cells expressing the fusion protein displayed a fluorescence pattern that included numerous, small punctate structures (Figure 1A) that were presumed to be peroxisomes. These were similar in appearance to the punctate structures observed in cells expressing a peroxisomal marker protein consisting of GFP appended to a carboxyl-terminal PTS1 ([23], GFP-SRM, Figure 1B). In addition, GFP-MFP transformed cells often displayed less intense, uniformly labeled filamentous structures that were concentrated in the cell cortex and were presumed to correspond to MTs (Figure 1A, inset). In contrast, cells expressing GFP alone displayed only diffuse nuclear and cytosolic fluorescence, as expected (Figure 1C).

**Figure 2:**
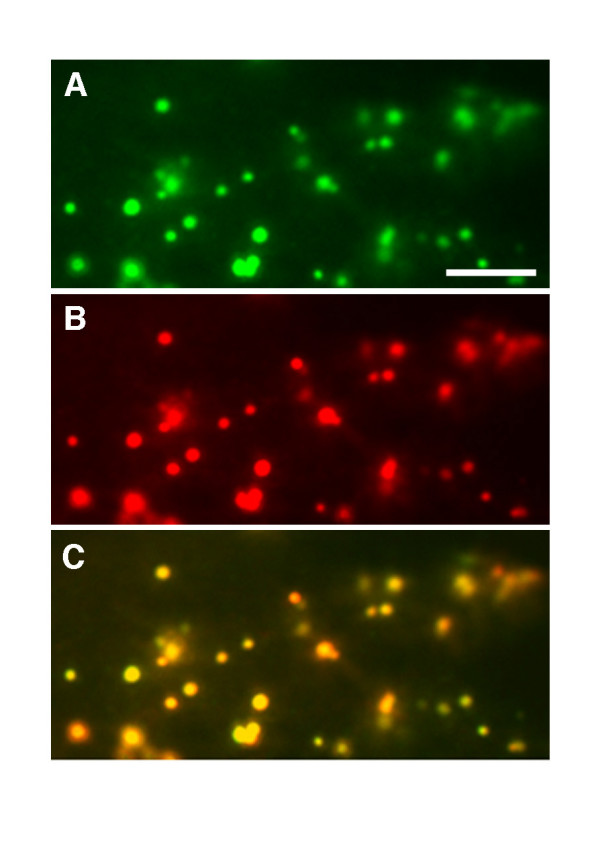
**GFP-MFP partially co-localizes with the endogenous peroxisomal matrix protein catalase**. Onion epidermal cells expressing GFP-MFP were fixed and then probed with monoclonal antibodies raised against catalase. GFP-MFP fluorescence (A), mouse anti-catalase antibody staining (B), and an overlay of the two images (C) are shown in a region of a single transformed cell by epifluorescence microscopy. The acquisition of these images was set at low exposure to capture the brightly fluorescing peroxisomes. The less intensely fluorescing MTs in this GFP-MFP expressing cell were not visible at this exposure. Bar, 4 μm.

**Figure 3:**
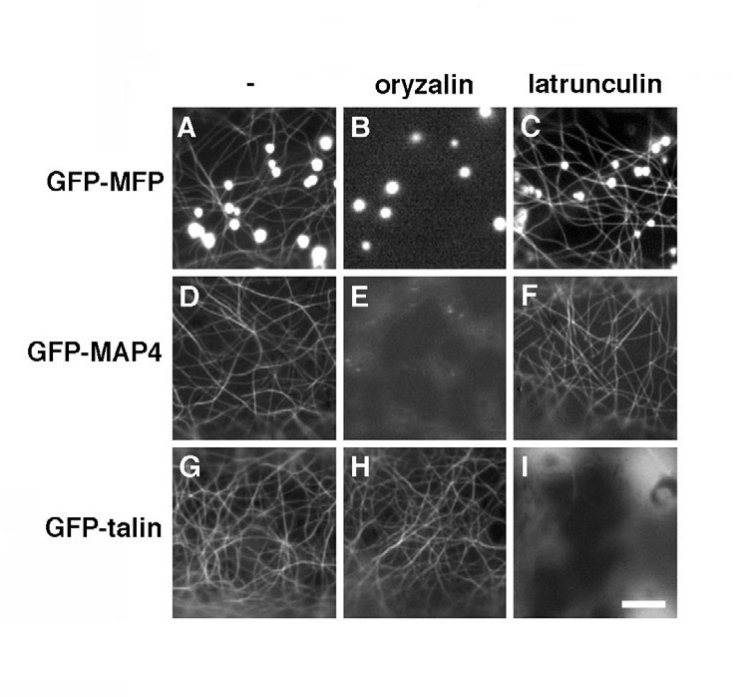
**The filamentous labeling in GFP-MFP-expressing cells is abolished when cells are treated with the MT-disrupting agent oryzalin**. Onion epidermal cells expressing GFP-MFP (A, B, C), GFP-MAP4 (D, E, F) or GFP-talin (G, H, I) were treated with DMSO (A, D, G), oryzalin (B, E, H) or latrunculin B (C, F, I) for 45 minutes and visualized by epifluorescence microscopy. Bar, 3 μm.

Indirect immunofluorescence and cytoskeleton-depolymerizing drug treatment experiments were conducted to determine if the punctate and filamentous structures identified in cells expressing GFP-MFP (Figure 1A) did indeed correspond to peroxisomes and MTs, respectively. Antibodies raised against the peroxisome matrix marker enzyme catalase were used to label peroxisomes in chemically fixed onion epidermal peels bombarded with the GFP-MFP fusion construct. Figure 2 shows that the punctate structures in GFP-MFP-expressing cells clearly co-localized with endogenous anti-catalase antibody labeling in the same cells, confirming that at least a portion of GFP-MFP was localized to peroxisomes. To confirm also that the filamentous structures labeled by GFP-MFP were MTs, GFP-MFP-transformed cells that displayed strongly fluorescing filaments were treated with reagents that cause cytoskeleton depolymerization. Figure 3A and 3B show that a 45 min treatment with the MT depolymerizing drug oryzalin specifically disrupted the filamentous structures in GFP-MFP expressing cells. As expected, MTs in cells expressing the MT-binding protein GFP-MAP4 [24] were also disrupted by oryzalin (compare Figures 3D and 3E). Conversely, treatments with latrunculin B, a potent inhibitor of actin filament assembly, had no effect on the stability of the filamentous structures in GFP-MFP (or GFP-MAP4) expressing cells (compare Figures 3A and 3C; 3D and 3F). However, latrunculin B treatment did effectively disrupt actin filaments in cells expressing a GFP-talin fusion protein [25] (compare Figures 3G and 3I). These results indicated that the labeled filaments observed in GFP-MFP transformed onion epidermal cells were MTs.

**Figure 4:**
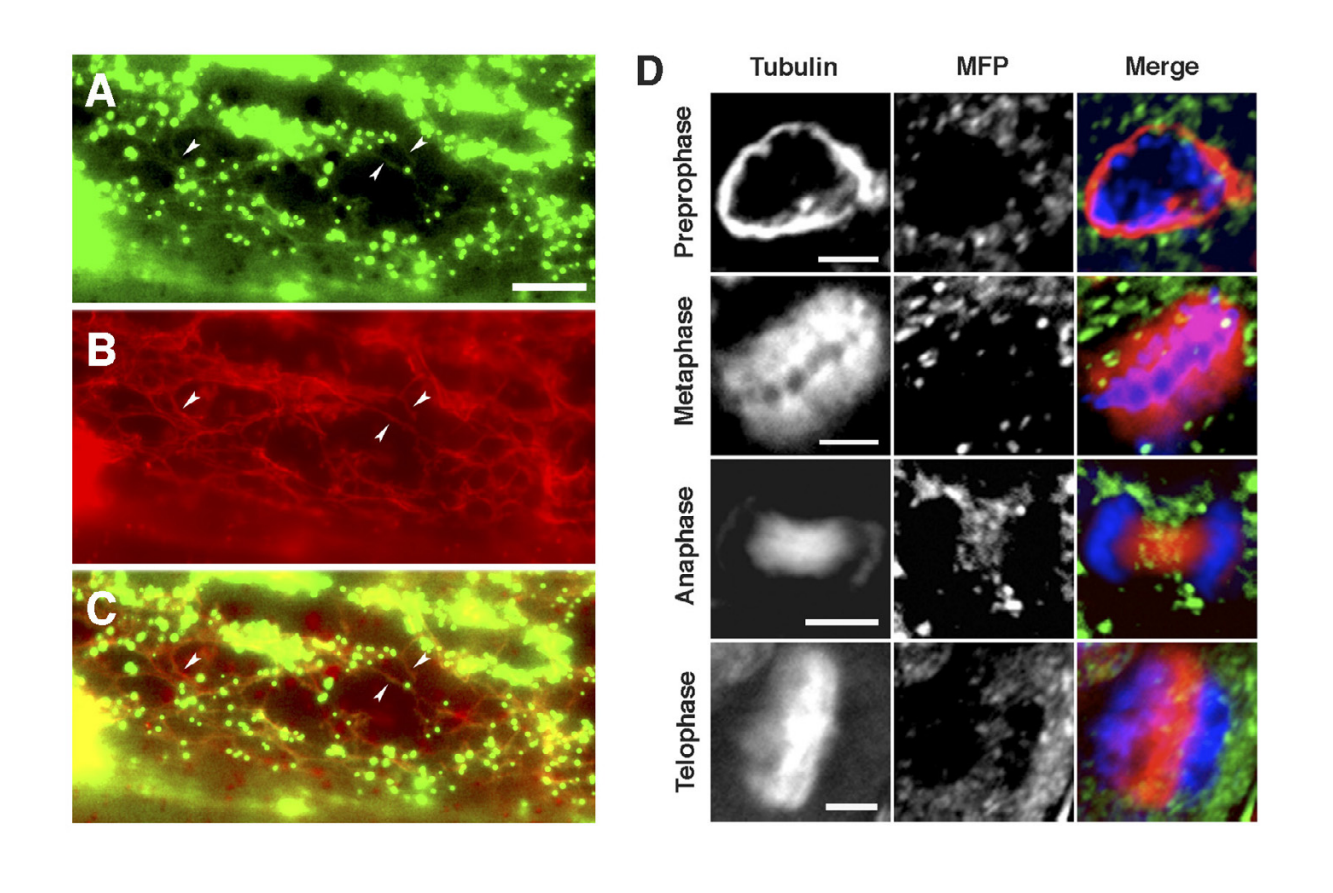
**Immunolocalization of MFP labels faint filaments that co-localize with cortical MTs, but does not label mitotic MT structures**. Indirect immunofluorescence analysis of a fixed onion epidermal cell probed with affinity purified rabbit anti-MFP antibodies (A) and mouse anti-tubulin antibodies (B) and visualized by epifluorescence microscopy. An overlay (C) shows the co-localization of MT labeling by the two antibodies (arrowheads). (D) Fluorescence immunostaining of MT structures with a tubulin antibody in four stages of mitosis in Arabidopsis suspension cells labels the pre-prophase band, the mitotic spindle in metaphase and anaphase, and the phragmoplast in telophase. The MFP antibody labels peroxisomes but not these MT structures in dividing cells. DNA was stained with DAPI (blue). Bars, 6 μm.

To determine if endogenous onion MFP also interacts with MTs and that the interaction of GFP-MFP to MTs described above was not a consequence of its transient over expression, we performed co-immunolocalization experiments using rabbit anti-MFP and mouse anti-tubulin antibodies. In addition to labeling peroxisomes, anti-MFP antibodies also labeled faint filamentous structures that corresponded to cortical MTs (Figure 4). We also performed immunolocalization experiments using Arabidopsis suspension culture cells in order to determine if MFP binds to the MT arrays that form during plant mitosis. These mitotic MT arrays include the pre-prophase band of MTs, the mitotic spindle, and the cytokinetic phragmoplast. Suspension culture cells are useful for observing these arrays since they divide rapidly during their logarithmic growth phase. Anti-MFP antibody labeling of cortical MTs was evident in non-dividing cells, similar to that observed in onion cells (not shown). However, we did not observe any fluorescence attributable to MFP binding to the mitotic MT arrays, as only peroxisome-specific fluorescence was visible in dividing cells (Figure 4D).

### Multiple MT-binding regions are present on the MFP polypeptide

**Figure 5:**
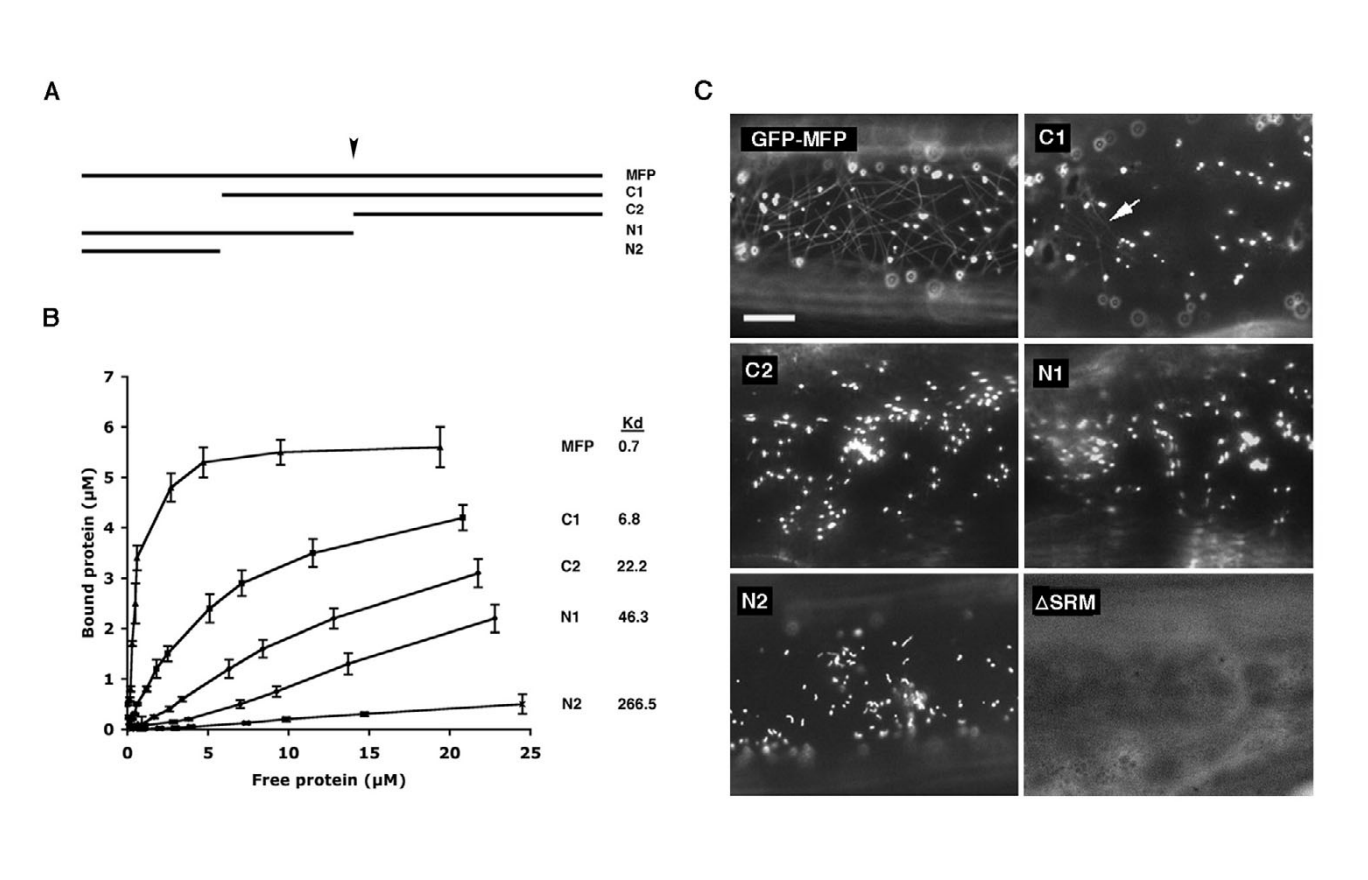
**Binding affinity of various truncated MFP polypeptides to MTs**. (A) Polypeptide map of wild type MFP and four deletion polypeptides of MFP (C1, C2, N1, N2) used for the expression of recombinant proteins in *E. coli *and GFP chimeras in onion epidermal cells. Arrow indicates the location of the putative KXGS MT-binding domain. This putative domain is disrupted between the leucine and valine residues (see Results) in the truncated polypeptides C2 and N1. (B) The recombinant proteins were used in MT co-sedimentation assays to generate MT-binding curves. Increasing concentrations of each truncated protein were used (0.25, 0.5, 0.75, 1.0, 2.0, 3.0, 4.0, 7.5, 10.0, 15.0, and 25.0 μM) with a constant amount of MTs (5 μM tubulin). Dissociation constant values (K_d_, μM) generated from the data in each of the curves are shown. (C) The subcellular localization pattern of the full-length MFP (GFP-MFP), truncated versions of MFP (C1, C2, N1, N2), and MFP lacking the PTS1 tripeptide (ΔSRM) fused to the carboxyl-terminus of GFP in onion epidermal cells. Faint MTs were occasionally evident in cells expressing the GFP-C1 fusion protein (arrow). Each image is a selected region from an individual cell. Bar, 10 μm.

In an effort to identify the region(s) of MFP that is responsible for binding to MTs we generated four truncated versions of recombinant MFP (Figure 5A) and determined their affinity for MTs using MT co-sedimentation assays. Two of the truncated polypeptides, C2 and N1, contained deletions that terminated within an internal region of MFP that is similar in sequence to a MT-binding domain found in several well-characterized MT-associated proteins including MAP2, MAP4, tau and TOGp [26]. The consensus sequence of this domain consists of the amino acid residues, -LX_5_KI/VGSE/DNK-, and is defined by a characteristic serine phosphorylation tetrapeptide motif (-KXGS-) known to regulate the MT-binding activity of this domain [26]. The amino acid sequence of the potential MT-binding site in MFP (-LEGLVKRGSLTKDK-) begins at amino acid position 356. We were unable to obtain a more detailed deletion analysis of the carboxyl-terminus of the protein (Figure 5A), as repeated efforts to express truncated versions of the protein in this region were not successful.

Figure 5B shows that full-length MFP had a high affinity for MTs as indicated by its dissociation constant (K_d_) value of 0.7 μM, a value similar to what we determined previously for the same protein (K_d _= 0.8 μM, [19]). In contrast, each of the amino- and carboxyl-terminal deletions of the MFP polypeptide (C1, C2, N1 and N2) displayed a significantly reduced affinity for MTs when compared to the full-length polypeptide. None of these deletions, however, resulted the complete elimination of MT-binding activity. The amino-terminal deletion polypeptide, C1, had the highest affinity for MTs (K_d _= 6.8 μM, Figure 5B) when compared to the other truncated proteins although its affinity for MTs was 10-fold less than the full-length polypeptide. Polypeptide N2 was composed of the region of MFP that was deleted in polypeptide C1 yet its affinity for MTs was the lowest of all the truncated polypeptides (K_d _= 266.5 μM). This indicates that this region alone does not possess significant binding activity, but has an important contribution to the overall binding activity of the full-length MFP. Polypeptides C2 (K_d _= 22.2 μM) and N1 (K_d _= 46.3 μM), on the other hand, were intermediate in their MT-binding activities despite being disrupted in the putative KXGS MT-binding domain. Overall, this deletion analysis indicates that there are multiple MT-binding regions located on the MFP polypeptide, and that they work synergistically to achieve the high MT-binding affinity observed for full-length MFP.

We performed next a series of transient expression experiments with the same truncated versions of MFP by expressing them in onion epidermal cells as fusions to the carboxyl-terminus of GFP (e.g., GFP-C1, Figure 5C). The objective of these experiments was to determine whether their relative MT-binding affinities as determined by MT co-sedimentation assays (Figure 5B) corresponded to their *in vivo *MT-binding characteristics. For those fusion constructs that lacked portions of the carboxyl-terminus of MFP (i.e., N1 and N2, Figure 5A), the endogenous MFP PTS1 sequence (-SRM) was restored so that these deleted polypeptides would retain their targeting to peroxisomes. All fusion constructs, along with wild-type GFP-MFP, were bombarded individually into onion epidermal cells and observed throughout a 3 to 24 hour period. While cells expressing full-length GFP-MFP often displayed MT labeling, the only truncated MFP fusion protein to label MTs was the amino-terminal deletion construct C1 (GFP-C1; Figure 5C). The number of GFP-C1-transformed cells displaying MT labeling, however, was low, as was the fluorescence intensity of the MTs in these cells. Nevertheless, that GFP-C1 localized to MTs was consistent with the observation that this polypeptide possessed the highest MT-binding affinity of the truncated recombinant proteins (Figure 5B). While cells expressing the N2, N1 and C2 fusion proteins did not display MT fluorescence, it is possible that these proteins bound to MTs *in vivo *at levels too low to be detected using our imaging system.

We hypothesized earlier [10,19] that the MT-binding activity of MFP may serve to concentrate MFP on MTs prior to its import into peroxisomes. Since the carboxyl-terminal PTS1 tripeptide (SRM) of MFP is both necessary and sufficient for import into peroxisomes (R.T. Mullen and D.G. Muench, unpublished observations), we anticipated that deletion of this sequence from MFP in the context of the GFP-MFP chimera would cause an accumulation of the protein in the cytosol and a resulting increase in binding to MTs. Surprisingly, expression of this modified chimera (i.e., GFP-MFPΔSRM) in onion epidermal cells resulted in only a diffuse cytosolic fluorescence with no detectable filamentous structures (Figure 5C). This apparent necessity of the MFP PTS1 for high affinity MT-binding *in vivo *further demonstrates the complex nature of the MT-binding activity of MFP and the requirement for multiple regions of the protein to achieve efficient binding.

### Confocal microscopy and real-time imaging indicate that peroxisomes and MTs interact frequently

**Figure 6:**
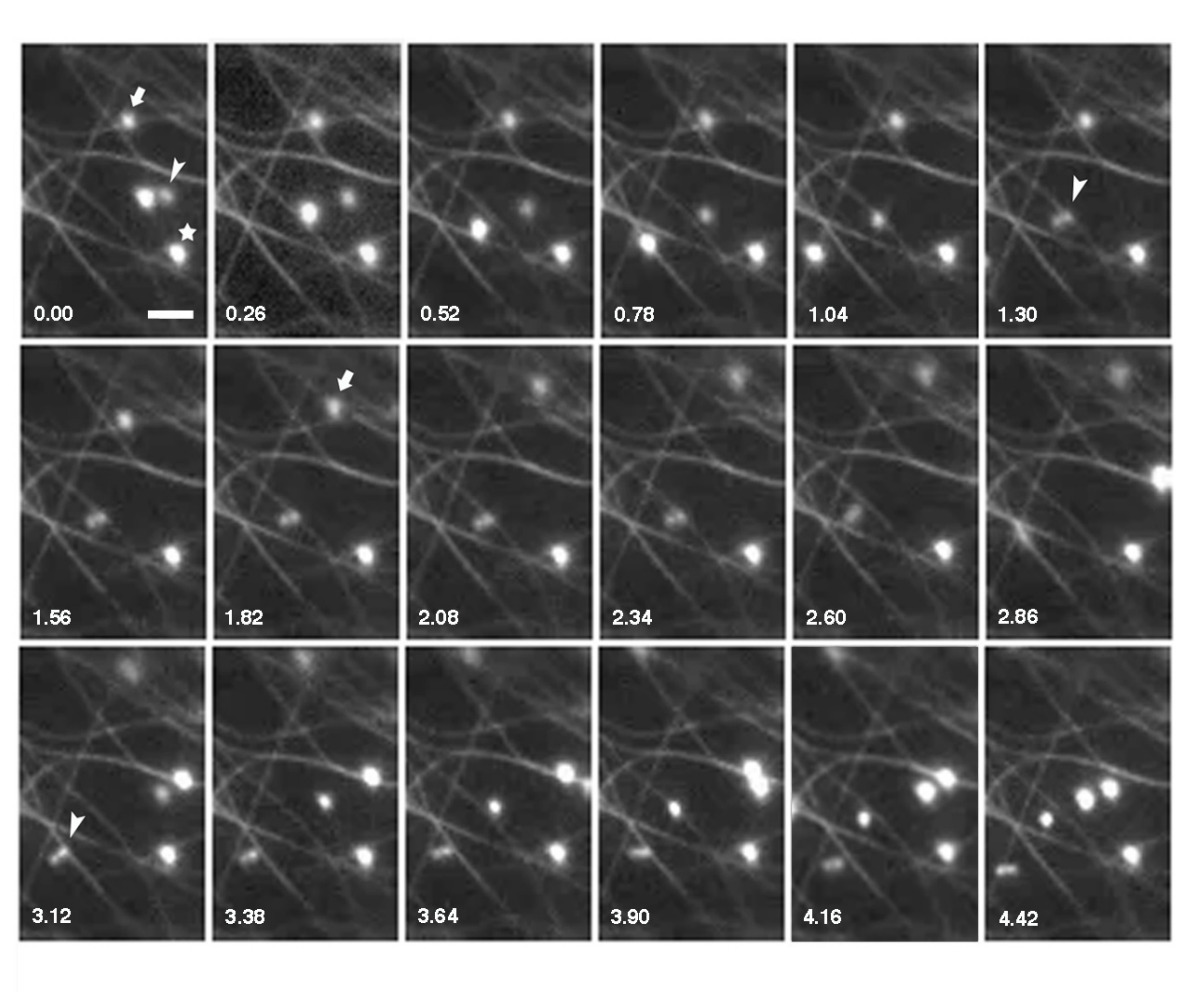
**Interactions are apparent between peroxisomes and MTs in onion epidermal cells**. Time-lapse images of a region from a GFP-MFP-expressing onion epidermal cell showing apparent transient and long-term interactions of peroxisomes and MTs. The movements of three peroxisomes were followed over 18 image frames. One of these peroxisomes (star) showed, throughout the entire series, oscillatory movements at a fixed location that were co-incident with a MT. Another peroxisome (arrow) remained fixed at a site coincident with a MT for approximately two seconds at the beginning of the image sequence, and then moved out of the field of view during the final two seconds. The initial movement is marked by the arrow in image frame 1.82. A dumbbell-shaped peroxisome (arrowhead) moved through the cytosol and appeared to transiently tether to a MT (second arrowhead, image frame 1.30). This peroxisome then released and tethered to another MT (third arrowhead, image frame 3.12) before releasing again and continuing its movement. The cell observed in this image sequence was from an unpeeled epidermal layer that remained associated with the leaf segment. The movements of the peroxisomes shown in this figure are also shown in a real-time image sequence in Additional data file Movie 1. Numbers indicate elapsed time in seconds. Bar, 2 μm.

Previous live-cell microscopy studies revealed that plant peroxisomes exhibit high velocity unidirectional movements, bi-directional and stop-and-go movements, as well as rapid oscillations at fixed locations within the cell, and that these movements occurred on actin filaments with no apparent role for MTs (reviewed in [10]). Using confocal microscopy of GFP-MFP-expressing onion epidermal cells we observed that peroxisomes and cortical MTs were regularly located in the same optical section (Figure 1A, inset). This indicated that peroxisomes are frequently in close association with MTs as they move through the cell cortex on actin filaments. In addition, we noted in real-time movies that the characteristic stop-and-go (or "pausing") of peroxisomes frequently occurred at sites occupied by MTs (Figure 6, see Additional data file 1: Movie 1, and Additional data file 2: Movie2), also suggesting that these two structures interact. We determined the frequency of these peroxisome pausing events on MTs in cells that had a relatively sparse cortical MT network such as those observed in Additional data files Movie 1 and Movie 2. The stop-and-go movements of peroxisomes (n = 246) from 14 GFP-MFP-expressing cells were observed in movies of 15 to 20 seconds duration. We determined that 67 ± 13% of the pausing events occurred at sites that were coincident with MTs. There were also numerous peroxisomes that exhibited oscillatory movements at fixed locations. These were observed to be in frequent contact with cortical MTs during their movements 73 ± 19% of the time. Notably, while treatment of GFP-MFP-expressing cells with latrunculin B for one hour caused peroxisomes to oscillate at fixed locations within the cell [16,17,27,28], treatment with oryzalin for one hour did not have an observable effect on the frequency of peroxisome pausing events associated with motile peroxisomes (see Additional data file 3: Movie3). This suggests that MTs are not directly involved in a majority of the peroxisomal pausing events, but that another mechanism is responsible for these pausing events.

Interestingly, we observed also that when epidermal peels were treated with oryzalin or latrunculin B immediately following bombardment of the GFP-MFP fusion construct and throughout the incubation period (see Materials and Methods for details about long-term drug treatments), there was a subtle effect on the rate of GFP-MFP labeling of peroxisomes compared to cells from untreated peels. The average frequency of cells showing peroxisome fluorescence 3 to 4 hours after bombardment in untreated peel segments (normalized value of 0.81 ± 0.08) was higher than in segments treated with oryzalin (0.65 ± 0.11) or latrunculin B (0.74 ± 0.08). These data indicate that the there is a reduction in the rate of synthesis and/or import of GFP-MFP as a result of cytoskeleton disruption.

## Discussion

The processes involved in the import of proteins into the peroxisome matrix are distinct from those described for protein import into other membrane-bound organelles. One major difference is that peroxisomal matrix proteins can be imported fully folded and/or in an oligomerized conformation [6,29,30]. This characteristic implies that these proteins have the potential to possess functional activities in the cytosol prior to their import. We reported previously that the plant peroxisomal MFP binds to MTs and RNA *in vitro*, in addition to possessing enzymatic activities related to fatty acid β-oxidation. We suggested that the MT-binding activity may function to facilitate the import of MFP into peroxisomes [10,19]. In this paper, we used indirect immunofluorescence microscopy and the expression of a GFP-MFP chimera to show that MFP interacts uniformly with interphase cortical MTs *in vivo *(Figures 1 and 4), thereby demonstrating that MFP is a *bona fide *MT-binding protein. The specific binding of MFP to the cortical MTs implies that the MFP-MT interaction is limited to interphase cells. Array-specific binding by other MT-binding proteins has been observed previously [31-33]. These specific interactions may be regulated by post-translational modifications such as phosphorylation/dephosphorylation of the MT-binding protein or modifications to the acidic carboxyl-terminal tail of tubulin [33,34].

Although the techniques used here showed that MFP binds to MTs *in vivo*, the level of detection of this interaction varied. Indirect immunofluorescence microscopy using an affinity-purified anti-MFP antibody labeled peroxisomes intensely, whereas MTs possessed only weak immunofluorescence and were often not visible at all. Similarly, many transformed cells expressing GFP-MFP lacked observable MT labeling, or MTs in these cells were visible only after computer software enhancement of digitized images. However, in numerous other bombardment experiments MTs were readily observable in up to one-third of the transformed cells. We were unable to determine whether this variation in MT labeling by GFP-MFP was due to differences in the level of expression of the fusion protein, the metabolic state of the cell, or the quality of the leaf material used in individual bombardment experiments.

MT co-sedimentation experiments of truncated versions of MFP indicated that there are multiple MT-binding domains within the MFP polypeptide (Figure 5). Each of the truncations resulted in a significant decrease in MT-binding affinity of the remaining polypeptide, indicating that maximal binding of the full-length MFP likely depends on several points of contact to MTs, as has been suggested for other MT-binding proteins [32]. MT-binding domains have been identified for numerous proteins, some of which contain well characterized domains such as those found in the animal MAPs tau, MAP4, MAP2, and TOGp [26], whereas others possess less well known MT-binding domains that are not as well studied [35,36]. We identified by sequence analysis a putative MT-binding domain in MFP that showed sequence similarity to a domain centered around a KXGS motif present singly or in repeats in some animal MAPs [26,33]. However, this region does not contribute significantly to the MT-binding activity of MFP *in vitro *(Figure 5). We have demonstrated that the Arabidopsis homolog of the rice MFP also possesses MT-binding activity (SDX Chuong and DG Muench, unpublished observations). Despite sharing 82% amino acid similarity to the rice sequence, the Arabidopsis protein does not possess the putative KXGS motif region that is present in the rice MFP. This is consistent with the observed minimal influence of this motif on MT binding affinity *in vitro *(Figure 5).

Interestingly, transient expression of a modified version of GFP-MFP that lacked the MFP carboxyl-terminal PTS1 resulted in this fusion protein (GFP-MFPΔSRM) being localized exclusively to the cytosol, with no apparent labeling of MTs (Figure 5C). This indicates that the carboxyl terminus of MFP is also required for its efficient binding to MTs *in vivo*. The lack of MT binding by this fusion protein appears to be an effect of expression *in vivo *since recombinant MFP△SRM binds to MTs *in vitro *with an affinity that is similar to the full-length MFP protein (SDX Chuong and DG Muench, unpublished results). The PTS1 normally interacts with the peroxisomal import receptor, Pex5p, to facilitate the targeting of PTS1-bearing proteins from the cytosol to the peroxisome surface and possibly across the peroxisome membrane [6,37]. Pex5p is then recycled and is available to interact with other PTS1-containing proteins. The necessity of the PTS1 for efficient binding of MFP to MTs *in vivo *may, therefore, highlight a novel role for Pex5p at an early stage of peroxisome protein sorting.

The immunofluorescence and live-cell imaging data presented here support our previous hypothesis that was based on *in vitro *MT-binding assays [19] and stated that the interaction between MFP and MTs assists in the efficient and regulated sorting of MFP to peroxisomes [10]. That is, MFP-MT interactions serve to concentrate MFP in the cell cortex, a region of the cell where peroxisomes are abundant and are frequently moving along actin filaments. Using confocal microscopy, we observed here that peroxisomes and cortical MTs regularly occupied the same three-dimensional space in the cell cortex (Figure 1A, inset). This arrangement would provide frequent opportunities for motile peroxisomes to interact with MTs as peroxisomes move in the cell cortex, a premise that is supported by previous evidence for a direct association between cortical MTs and actin filaments [38]. In addition, real-time fluorescence imaging revealed apparent MT interactions with peroxisomes in the form of pausing events by motile peroxisomes as well as peroxisomes that oscillated at fixed locations (Figure 6, Movie Supplement 1). These types of peroxisome-MT interactions would readily allow for the transfer of MT-bound MFP to the import machinery located on the peroxisomal surface.

Additional support for a MT role in peroxisomal protein import comes from the discovery that six other plant peroxisomal matrix proteins also possess tubulin-binding activities [22,39]. Most of these proteins have activities involved in catalyzing the reactions of fatty acid β-oxidation in the peroxisome, however, one of them (malate dehydrogenase) is involved in the glyoxylate cycle. β-oxidation enzymes presumably do not have a metabolic role in the cytosol, since their associated metabolic pathways have not been shown to exist in that compartment. It is more likely, therefore, that the binding of these peroxisomal matrix proteins to MTs functions in a general sorting pathway to the peroxisome, as discussed above for MFP.

If the cytoskeleton is required for the efficient delivery of MFP to the peroxisome surface as described above, then treatment of cells with cytoskeleton destabilizing agents should affect the import of MFP into peroxisomes. We determined that long-term treatments of epidermal peels with oryzalin and latrunculin B immediately after bombardment of GFP-MFP resulted in a small reduction in the rate of fluorescence accumulation by peroxisomes, indicating that at least subtle roles exist for the cytoskeleton in MFP import. However, since our transient expression system involved an over-expression of GFP-MFP, this may have overridden any requirement for MTs without significantly affecting protein import. We are currently conducting detailed experiments that are beyond the scope of the present study which utilize alternative approaches to determine a potential role of the cytoskeleton on MFP sorting to the peroxisome.

## Conclusion

These data provide conclusive evidence for an interaction between peroxisomal MFP and MTs in plant cells, and revealed that this association is specific to the cortical MT array. Confocal and live cell imaging indicated also that interactions between peroxisomes and MTs occur frequently in the cell cortex. These observations reinforce our hypothesis for a MT role in the sorting of MFP to the peroxisome.

## Methods

### GFP expression constructs and biolistic transformation of onion epidermal cells

Full-length and truncated versions of a rice peroxisomal MFP cDNA (Genbank accession C26129, [19]) were synthesized by PCR amplification for the production of GFP fusion constructs (Figure 5A) using the following oligonucleotide primers:

gMFP1 – CACTCTAGAATGGCGGGGGCGATCCGCGTCACC; gMFP10 – CGTCTAGATCACATGCGTGACCTCGA; gMFP11 – CGTCTAGATCACATGCGTGAAAGACCACCTTCTTTC; gMFP12 – GCGTCTAGATCACATGCGTGAGACTAGACCCTCAAGATTAG; gMFP14 – CACTCTAGAGTTGATGCCCTGTGCTCC; gMFP15 – CACTCTAGAGGCTCATTGACAAAGCAC; gMFP16 – CCATCAGCTGAAATAGCATCTAGAATGCGTTACCTCGACGAAGCCTGCTGG.

Primer pairs were used to amplify either full-length MFP (primers gMFP14 and gMFP10), the amino-terminal truncations C1 (primers gMFPl4 and gMFP10) and C2 (primers gMFP15 and gMFP10), the carboxyl-terminal truncations N1 (primers gMFP1 and gMFP11), N2 (primers gMFP1 and gMFP12), or GFP-MFP minus the PTS1 sequence (GFP-MFPΔSRM, primers gMFP1 and gMFP16). Each of the PCR primers contained an *Xba*I sequence in their 5' flanking region. Digestion of the PCR products with *Xba*I allowed for cloning into the *Xba*I site of pRTL2ΔNS/GFP-*Xba*I [40], resulting in an in-frame fusion of the full-length MFP or truncated MFP open reading frame to the carboxyl-terminus of GFP. pRTL2ΔNS/GFP-*Xba*I is a plant expression vector containing the cauliflower mosaic virus 35S promoter and nopaline synthase terminator, and a modified GFP with an in-frame *Xba*I site instead of a stop codon. GFP-talin [25] and GFP-MAP4 [24] expression constructs were used as controls in some transient expression experiments to label actin filaments and MTs, respectively.

Transient expression of the various GFP fusion constructs was achieved by biolistic bombardment into onion (*Allium cepa*) epidermal cells using protocols that were described previously [41]. Plasmid DNA was purified using a DNA Maxiprep kit (Qiagen, Mississauga, ON, Canada) and 5 to 10 μg of the DNA was coated onto gold particles (1 μm diameter) as described by the manufacturer (BioRad, Hercules, CA). DNA-coated gold particles were bombarded into the adaxial surface of onion bulb leaf segments (1 cm^2^) from a distance of 10 cm using a Biolistic PDS-1000/He particle delivery system (Bio-Rad) at a pressure of 1200 psi. After bombardment, the leaf sections were placed on moist filter paper in petri dishes, adaxial side down, and incubated in the dark at room temperature for 3 to 24 hours. Alternatively, the epidermal layer was peeled immediately after bombardment and floated on liquid MS media (pH 5.9) containing 3% sucrose, and peels were then placed in the dark at room temperature for 3 to 24 hours.

### Expression and purification of recombinant proteins and MT co-sedimentation assays

PCR was used to amplify full-length or truncated versions of the rice MFP coding region for the production of recombinant proteins (Figure 5A) using the following oligonucleotide primers:

rMFP1 – CACGGATCCGCGGGGGCGATCCGCGTCACCATG; rMFP10 – CACCTGCAGCATGCGTGACCTCGACGAAGCCTG; rMFP11 – GCGCTGCAGAAGACCACCTTCTTTCCCTTCCTT; rMFP12 – GCGCTGCAGGACTAGACCCTCAAGATTAGCTGC; rMFP14 – CACGGATCCGTTGATGCCCTGTGCTCCCCTGAT; rMFP15 – CACGGATCCAAGAGGGGCTCATTGACAAAGGAC. Primer pairs were used to amplify either full-length MFP (primers rMFP1 and rMFP10), the amino-terminal truncations C1 (primers rMFP14 and rMFP10) and C2 (primers rMFP15 and rMFP10), and the carboxyl-terminal truncations N1 (primers rMFP1 and rMFP11) or N2 (primers rMFP1 and rMFP12). Each primer contained either *Bam*HI or *Pst*I restriction enzyme sites that were compatible for cloning into the expression vector pQE30 (Qiagen). Recombinant proteins were expressed in *E. coli *and purified as described previously [19]. The purified recombinant proteins were used in bovine MT co-sedimentation assays to generate binding curves for the determination of MT/MFP dissociation constants (K_d_) [19]. The co-sedimentation assays were repeated twice for each of the recombinant MFPs, and their mean values and standard deviations were presented. BSA was used as an internal negative control in selected MT co-sedimentation assays to ensure that soluble protein was not trapped by MTs in the pellet fraction. The K_d _value for each recombinant protein was determined using the Prism kinetics program (GraphPad Prism, Version 3.02, GraphPad Software Inc., San Diego, CA).

### Affinity purification of antibodies and immunolocalizations

Two mg of the full-length recombinant MFP was covalently coupled to 1 mL of a matrix consisting of an equal mixture of Affigel-15 (Bio-Rad) and Sepharose CL-6B (Sigma-Aldrich Ltd., Oakville, Canada), and the matrix was then poured into a disposable 2 mL column (Poly Prep, BioRad). One mL of MFP antiserum [19] was added to the column matrix, incubated overnight at 4°C, and the matrix was then washed with 5 column volumes of phosphate buffered saline (PBS). IgGs were eluted from the column with 2 mL of 100 mM glycine, pH 2.5, and the eluent was immediately neutralized by the addition of one-tenth volume 1.5 M Tris-HCl pH 8.8. Purified IgGs were concentrated using a Centricon-10 microfiltration apparatus (Amicon, Millipore, Billerica. MA) and stored at 4°C in the presence of 0.05% (w/v) sodium azide.

Onion epidermal peels were fixed for 5 min in freshly prepared paraformaldehyde (7.4% [w/v], Sigma-Aldrich Ltd.) and glutaraldehyde (0.015% [w/v], Sigma-Aldrich Ltd.) in a cytoskeleton-stabilizing buffer (50 mM HEPES pH 6.9, 2 mM MgSO_4_, 5 mM EGTA, and 0.1% Triton X-100)[42]. After fixing, the peels were washed three times in PBS for 5 min each, followed by treatment with 0.5% cellulase (Onazuka R-10) and 0.05% pectolyase Y23 (Seishin Pharmaceutical Co., Tokyo) in MS media (pH 5.9) containing 0.3 M mannitol for 3 minutes at 37°C. The peels were washed three times in PBS for 5 min each and then treated with 1% Triton-X 100 for 5 min at room temperature. Arabidopsis suspension cells were used for immunolocalization studies three days after subculturing. One mL of cell culture was fixed for 15 min at room temperature in freshly prepared 4% paraformaldehyde dissolved in cytoskeleton-stabilizing buffer. After fixing, the cells were washed three times with PBS for 5 min each, and then digested with 0.1% pectolyase Y-23 in PBS for 15 min at 30°C. The cells were washed three times in PBS for 5 min each and then treated with 1% Triton-X 100 for 5 min at room temperature.

The fixed and permeabilized epidermal peels and suspension cells were incubated for 15 min at room temperature in blocking solution (PBS containing 3% BSA), followed by incubation for 60 min in blocking buffer containing the appropriate primary antibodies. The following primary antibodies were used: affinity purified rabbit anti-MFP IgGs ([19], final concentration, 0.5 μg/mL); mouse anti-tobacco catalase (undiluted hybridoma medium purchased from the Princeton Monoclonal Antibody Facility); and mouse anti-α-tubulin monoclonal antibody (Sigma-Aldrich Ltd.), 1:500 (v/v). After three, 5 min washes in PBS, the peels/cells were incubated in secondary antibodies diluted 1:500 for 30 min at room temperature, and then washed again in PBS. The secondary antibodies used were goat anti-rabbit IgG-Alexa-594 conjugate and goat anti-mouse IgG-Alexa-488 conjugate (Molecular Probes, Eugene, OR). Pre-incubation of anti-MFP antibodies with recombinant MFP protein prior to using the antibodies in immunolocalization experiments was used to confirm the specific labeling of the anti-MFP antibodies.

### Microscopic analysis

Unpeeled onion leaf segments were secured to a glass slide with 1% agarose, and the epidermal cells were observed through a bead of water or MS media using a 63× long working distance water immersion lens (HCX Apo L, Leica, Germany) mounted on an epifluorescence microscope (Leica DMR). Alternatively, fresh or fixed epidermal peels were mounted on a glass slide in MS media or PBS, covered with a coverglass, and observed using either a Plan Fluotar 40× or Plan Apo 63× oil immersion objective lens (Leica). Images were captured using a cooled CCD camera (Retica 1350 EX, QImaging, Burnaby, BC, Canada) through the FITC or rhodamine filter sets. Image enhancement and deconvolution confocal algorithm manipulations were performed using Openlab software (Version 3.0, Improvision, Lexington, MA). Quicktime movies were generated from sequential images using the Openlab software, and single images were modified using Adobe Photoshop image processing software (Version 8.0, Adobe Systems Inc., San Jose, CA). The frequency of peroxisome pausing events on MTs was determined from 14 GFP-MFP expressing cells, and variation between cells was reported as standard deviation from the mean.

### Cytoskeleton drug treatments

Short-term cytoskeleton drug treatments of 45 min to one hour were conducted on transiently transformed onion epidermal layers that were bombarded with the GFP-MFP construct 3 to 24 hours in advance, and that possessed numerous cells showing high levels of fluorescence. Final drug concentrations were 0.2 μM latrunculin B (Sigma-Aldrich Ltd.) or 2 μM oryzalin (Crescent Chemical Company, Islandia, NY). Stock solutions of these drugs were prepared in DMSO. Epidermal peels were treated with the drugs by floating the peels on liquid MS media (pH 5.9) containing 3% sucrose and the appropriate amount of DMSO or drug. To demonstrate that these drug concentrations were effective in MT and actin filament disruption, epidermal cells expressing the GFP-MAP4 or GFP-talin constructs were treated with the corresponding drugs.

For experiments performed to determine the long-term effect of cytoskeleton disrupting drugs on GFP-MFP import into peroxisomes, each bombarded epidermal peel was divided equally into thirds, and the resulting sections were then treated individually with DMSO, oryzalin or latrunculin B immediately after bombardment by floating the peels on liquid MS media containing the appropriate amount of DMSO or drug. The bombarded epidermal peels were observed by epifluorescence microscopy, and the number of cells showing peroxisome labeling were determined 3 to 4 hours post-bombardment, since control sections typically showed the first signs of fluorescence during this period. Data from sections derived from individual peels were pooled for each experiment, and the total dataset presented included results from at least three individual experiments. Normalization of the raw data was performed, since the total number of transformed cells varied between individual bombardment experiments. Variation between experiments was reported as standard deviation from the mean.

## Authors' contributions

SDXC contributed to the experimental design, conducted a majority of the experiments, and assisted in manuscript writing, data analysis, and figure preparation. N-IP and MCF assisted with the immunofluorescence and transient expression experiments. RTM provided expression vectors, assisted in the transient expression experiments, and contributed to the experimental design and writing of the manuscript. DGM wrote the original draft of the manuscript, contributed to the experimental design, and assisted with the immunofluorescence and transient expression experiments, data analysis, and figure preparation. All authors read and approved the final manuscript.

## Supplementary Material

Additional data file 1 **Movie1.mov – Real-time image sequence of an onion epidermal cell expressing GFP-MFP shows apparent interactions between peroxisomes and cortical MTs**. Epifluorescence images (n = 200) were captured every 0.13 seconds. A mobile, dumbbell-shaped peroxisome (tracked with the green marker) appears to interact with MTs at several locations during the initial part of the image sequence before moving out of focus. Upon returning into the focal plane near the end of the image sequence, this peroxisome again appears to interact with MTs. The movement of this peroxisome is also shown in the fixed image sequence in Figure 6. Another peroxisome demonstrated a pausing event at a location where MTs were absent (tracked with the red marker), although it also paused several times at locations that coincided with MTs. The peroxisome tracked with the blue marker demonstrated on two occasions a subtle, lateral gliding movement alongside MTs. The cell observed in this image sequence was from an unpeeled epidermal layer that remained associated with the leaf segment. Click here for file

Additional data file 2 **Movie2.mov – Real-time image sequence of an onion epidermal cell expressing GFP-MFP that demonstrated a lower frequency of peroxisome pausing events at sites that were coincident with MT locations**. Epifluorescence images (n = 270) were captured every 0.2 seconds. Click here for file

Additional data file 3 **Movie3.mov – Real-time image sequence of an oryzalin treated onion epidermal cell expressing GFP-MFP**. This movie demonstrates that MT depolymerization does not result in an obvious reduction of peroxisome pausing events in cell cortex. Epifluorescence images (n = 150) were collected every 0.2 seconds. Click here for file
